# A case of perihilar cholangiocarcinoma with bilateral ligamentum teres hepatis treated with hepatopancreatoduodenectomy

**DOI:** 10.1186/s40792-020-0793-4

**Published:** 2020-01-30

**Authors:** Fumihiro Terasaki, Yusuke Yamamoto, Teiichi Sugiura, Yukiyasu Okamura, Takaaki Ito, Ryo Ashida, Katsuhisa Ohgi, Shintaro Akamoto, Katsuhiko Uesaka

**Affiliations:** Division of Hepato-Biliary-Pancreatic Surgery, Shizuoka Cancer Center Hospital, 1007, Shimo-Nagakubo, Sunto-Nagaizumi, Shizuoka, 411-8777 Japan

**Keywords:** Bilateral ligamentum teres, Liver metastasis, Right-sided ligamentum teres, Hepatopancreatoduodenectomy

## Abstract

**Background:**

Bilateral ligamentum teres (BLT) hepatis is a very rare anomaly defined as the connection of the bilateral fetal umbilical veins to both sides of the paramedian trunk, and it has never been reported in the English literature.

**Case presentation:**

A 72-year-old man who presented with obstructive jaundice was referred to our hospital. Contrast-enhanced computed tomography revealed that the patient had right-sided ligamentum teres (RSLT) and left-sided ligamentum teres (LSLT). The umbilical portion of the left portal vein, which the LSLT connected, became relatively atrophic in this patient. The RSLT attached to the tip of the right anterior pedicle and formed the umbilical portion of the right portal vein. The patient was diagnosed with perihilar cholangiocarcinoma which had invaded the root of the posterior branch of the bile duct, LHD, and intrapancreatic bile duct. The central bisectionectomy, in which the liver parenchyma was resected along the RHV on the right side and the LSLT on the left side, and caudate lobectomy combined with pancreatoduodenectomy were performed.

The presence of the patient with BLT is important for ascertaining the mechanism of the development of RSLT. Two umbilical veins are present initially during the embryonic stage. In general, the right-sided vein disappears, and the atrophic left-sided vein remains connected to the left portal vein originating from the vitelline vein. Several papers on the mechanism of the development of RSLT have been published. Some authors have mentioned that a residue of the right umbilical vein and the disappearance of the left umbilical vein are the causes of RSLT. On the other hand, some authors have asserted that RSLT is the result of atrophy of the medial liver area. The presence of BLT in patients indicates that the mechanism of the development of RSLT is characterized by a residue of the right umbilical vein and the disappearance of the left umbilical vein.

**Conclusions:**

The mechanism and origin of RSLT can be understood through cases of BLT, and surgeons must pay attention to anomalies of the portal and hepatic veins in patients with abnormal ligamentum teres.

## Background

Bilateral ligamentum teres (BLT) hepatis is a very rare anomaly defined as the connection of the bilateral fetal umbilical veins to both sides of the paramedian trunk, and it has never been reported in the English literature.

## Case presentation

**Fig. 1:**
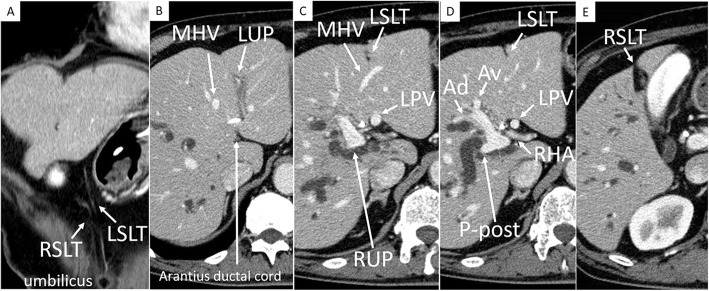
**a** Contrast-enhanced computed tomography (CT) image reveals that the patient had right-sided ligamentum teres (RSLT) and left-sided ligamentum teres (LSLT). **b** The umbilical portion of the left portal vein (LUP), to which LSLT connected, became atrophic in this patient. **c** The RSLT was attached to the tip of the right anterior pedicle and formed an umbilical portion of the right portal vein (RUP). **d** The right portal vein first ramified the posterior branch and formed the RUP, which ramified the anterior branch feeding the ventral side and the anterior branch feeding the dorsal side separately. **e** The RSLT was detected on the right side of the gallbladder. RSLT, right-sided ligamentum teres; LSLT, left-sided ligamentum teres; MHV, middle hepatic vein; LUP, the umbilical portion of the left portal vein; RUP, the umbilical portion of the right portal vein; LPV, left portal vein; RHA, right hepatic artery; Ad, the anterior portal vein branch feeding the dorsal side; Av, the anterior portal vein branch feeding the ventral side

**Fig. 2:**
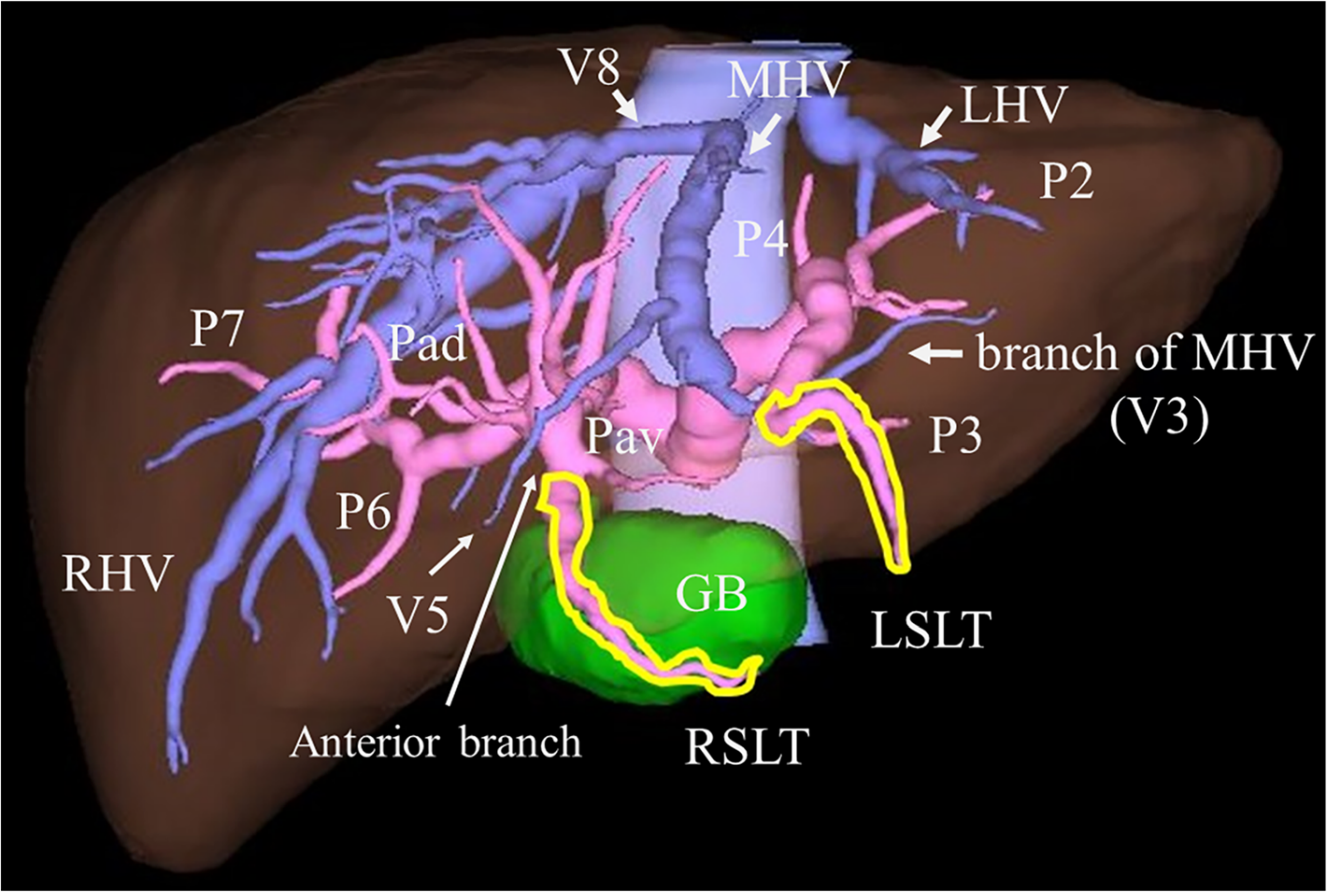
The three-dimensional CT image illustrated by a Synapse Vincent three-dimensional volume analyzer (Fujifilm Holdings Corporation, Tokyo, Japan) is shown. The middle hepatic vein (MHV) had shifted to the left side, and the branch of the MHV drained the anterior inferior and left paramedian sections as well as part of the left lateral inferior section. GB, gallbladder; LHV, left hepatic vein; LSLT, left-sided ligamentum teres; MHV, middle hepatic vein; RHV, right hepatic vein; RSLT, right-sided ligamentum teres; V3, lateral inferior branch of the hepatic vein; V5, anterior inferior branch of the hepatic vein; V8, anterior superior branch of the hepatic vein; Pad, anterior dorsal branch of the portal vein; Pav, anterior ventral branch of the portal vein; P2, lateral superior branch of the portal vein; P3, lateral inferior branch of the portal vein; P4, left medial branch of the portal vein; P5, anterior inferior branch of the portal vein; P6, posterior inferior branch of the portal vein; P7, posterior superior branch of the portal vein; P8, anterior superior branch of the portal vein

**Fig. 3:**
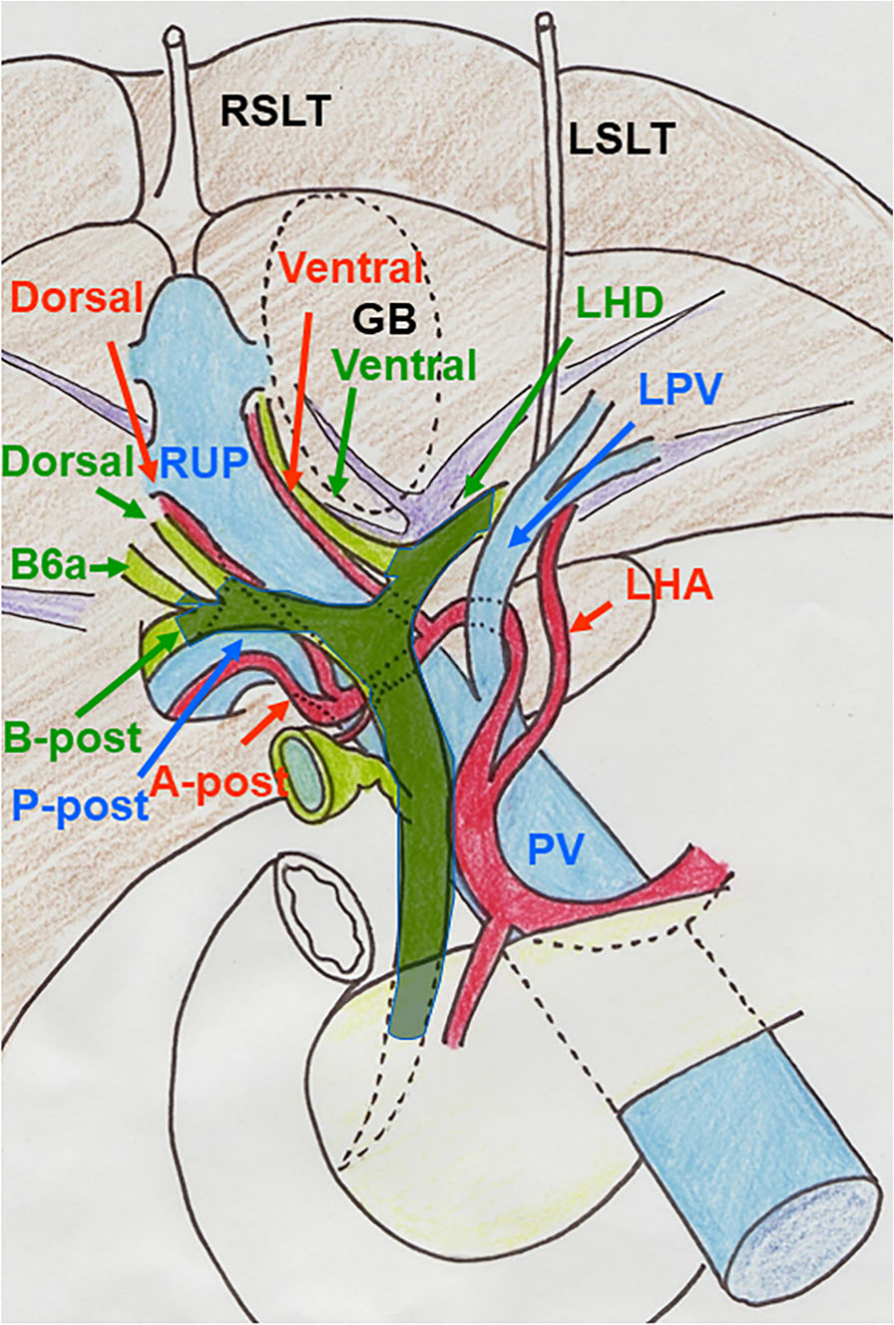
The preoperative schema of the perihilar anatomy and cancer progression is shown. The anterior branch of the hepatic artery feeding the ventral and dorsal sides ran along the RUP. The anterior branch of the bile duct drained the ventral and dorsal sides and ran along the RUP in this patient. A-post, posterior branch of the portal vein; B-post, posterior branch of the bile duct; B6a, posterior ventral branch of the bile duct; GB, gallbladder; LHA, left hepatic artery; LHD, left hepatic duct; LSLT, left-sided ligamentum teres; LPV, left portal vein; P-post, posterior branch of the portal vein; PV, portal vein; RSLT, right-sided ligamentum teres; RUP, the umbilical portion of the right portal vein

**Fig. 4:**
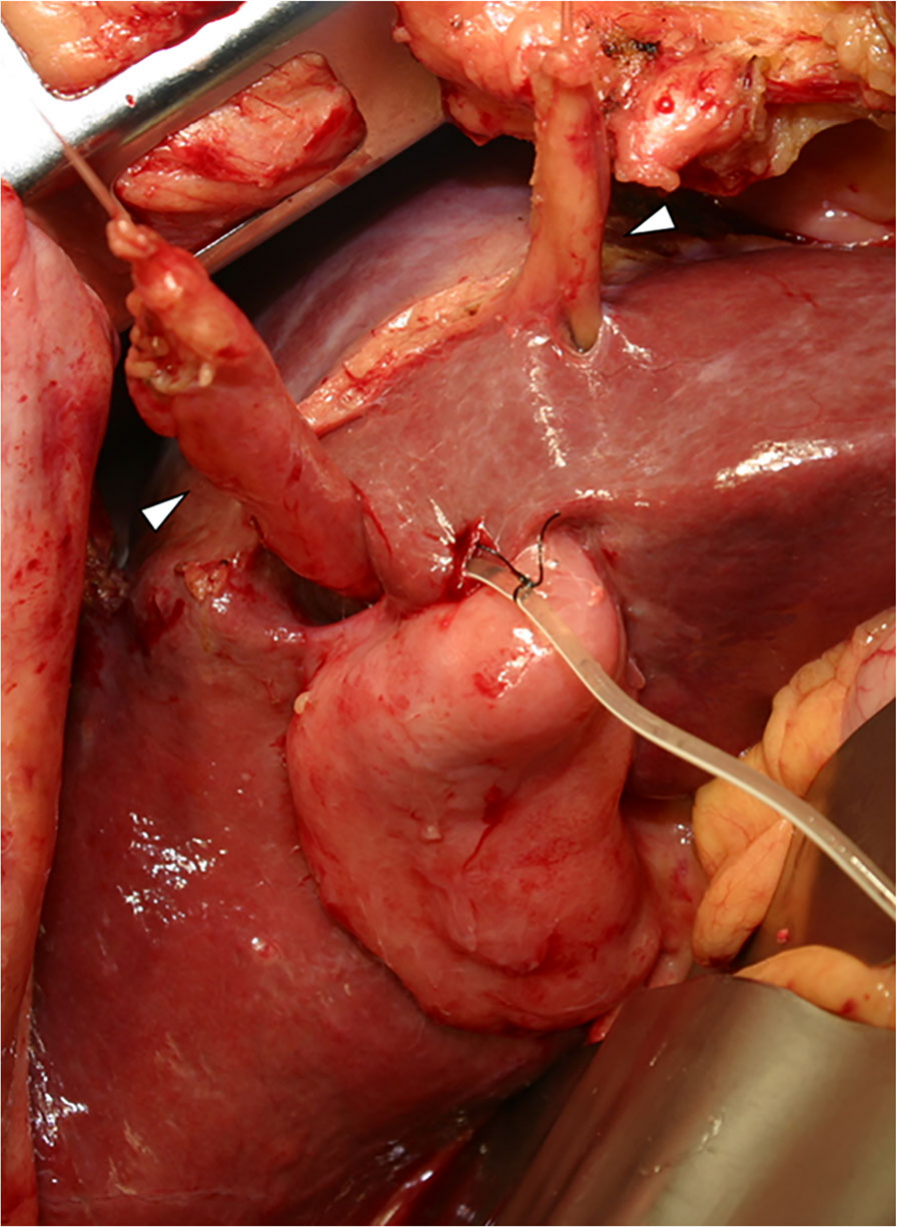
The RSLT connected to the RUP on the right side of the gallbladder, and the LSLT connected to the LUP on the left side of the gallbladder. The RSLT and LSLT were resected separately (arrow heads). LSLT, left-sided ligamentum teres; LUP, the umbilical portion of the left portal vein; RSLT, right-sided ligamentum teres; RUP, the umbilical portion of the right portal vein

A 72-year-old man who presented with obstructive jaundice was referred to our hospital. Contrast-enhanced computed tomography (CT) revealed that the patient had right-sided ligamentum teres (RSLT) and left-sided ligamentum teres (LSLT) (Fig. 1a). The umbilical portion of the left portal vein (LUP), which the LSLT connected, became relatively atrophic in this patient (Fig. 1b). The RSLT attached to the tip of the right anterior pedicle and formed an umbilical portion of the right portal vein (RUP) (Fig. 1c). The right portal vein first ramified the posterior branch and formed the RUP, which ramified the anterior branch feeding into the ventral side and the anterior branch feeding into the dorsal side separately (Fig. 1d). Another ligamentum teres, right-sided ligamentum teres (RSLT), was detected on the right side of the gallbladder (Fig. 1e). Three-dimensional CT images taken with a Synapse Vincent three-dimensional volume analyzer (Fujifilm Holdings Corporation, Tokyo, Japan) are shown in Fig. 2. The middle hepatic vein (MHV) had shifted to the left side, as has been reported previously in RSLT patients [1], and the branch of the MHV drained the anterior inferior and left paramedian sections as well as part of the left lateral inferior section. The patient was diagnosed with perihilar cholangiocarcinoma, and hepatopancreatoduodenectomy was planned after biliary drainage. The preoperative schema of the perihilar anatomy and cancer progression is shown in Fig. 3. The anterior branch of the hepatic artery feeding the ventral and dorsal sides ran along the RUP. The anterior branch of the bile duct drained the ventral and dorsal sides and ran along the RUP in this patient. The cholangiocarcinoma had invaded the root of the posterior branch of the bile duct, LHD, and intrapancreatic bile duct. The indocyanine green retention at 15 min was 10%. Child-Pugh classification was class A. The posterior section and lateral section accounted for 35.9% and 23% of total liver volume. Left trisectionectomy is still associated with high morbidity and mortality rate [2], and taking the risk of liver failure into consideration, we performed central bisectionectomy. Laparotomy revealed that the RSLT was connected to the RUP on the right side of the gallbladder, and the LSLT was connected to the LUP on the left side of the gallbladder. The RSLT and LSLT were resected separately (Fig. 4). The central bisectionectomy, in which the liver parenchyma was resected along the RHV on the right side and the LSLT on the left side, and caudate lobectomy combined with pancreatoduodenectomy were performed. The patient was discharged on postoperative day 66 after overcoming postoperative pancreatic fistula.

## Discussion

The ligamentum teres hepatis is a remnant of the umbilical vein that exists in the embryonic stage. The ligamentum teres hepatis connects the LUP and the Arantius duct (ligamentum venosum), which is an important landmark during liver dissection. Only one ligamentum teres hepatis exists in most cases, but we experienced a case of two ligamentum teres hepatis and demonstrated some concerns raised during surgical dissection; we also ascertained the mechanism of the development of RSLT, which exhibits a reported prevalence of 0.1–1.2% [3].

From the viewpoint of surgical resection, surgeons must pay attention to the anomalies of the portal and hepatic veins. In this case, the RSLT was attached to the tip of the right anterior pedicle and formed the RUP, and the LSLT formed the relatively atrophic LUP. The Arantius duct was continuous with the left portal vein. The middle hepatic vein (MHV) had shifted to the left side, and the branch of the MHV drained the anterior inferior and left paramedian sections as well as part of the left lateral inferior section. The artery and bile duct in this BLT patient ran along the portal vein similar to what is found in normal patients, though the anterior inferior branch and anterior superior branch of the artery and the bile duct ran beside the RUP. Preoperative simulation is important in patients with planned BLT hepatectomy, and surgeons should perform hepatectomy with consideration for these features.

The presence of the patient with BLT is also important for ascertaining the mechanism of the development of RSLT. The mechanism of the development of RSLT has been discussed in recent decades. Two umbilical veins are present initially during the embryonic stage. In general, the right-sided vein disappears, and the atrophic left-sided vein remains connected to the left portal vein originating from the vitelline vein [4]. Several papers on the mechanism of the development of RSLT have been published. Some authors have mentioned that a residue of the right umbilical vein and the disappearance of the left umbilical vein are the cause of RSLT [3]. On the other hand, some authors have asserted that RSLT is the result of atrophy of the medial liver area [5]. The presence of BLT in patients indicates that the mechanism of the development of RSLT is characterized by a residue of the right umbilical vein and the disappearance of the left umbilical vein.

## Conclusions

The mechanism and origin of RSLT can be understood through cases of BLT, and surgeons must pay attention to anomalies of the portal and hepatic veins in patients with abnormal ligamentum teres.

## Data Availability

The datasets supporting the conclusions of this article are included within the article and its additional files.
